# Identifying prenuptial births from family pedigrees using record linkage.

**DOI:** 10.23889/ijpds.v7i3.1784

**Published:** 2022-08-25

**Authors:** Nishadi Kirelle, Peter Christen, Eilidh Garrett

**Affiliations:** 1 School of Computing, The Australian National University; 2 University of Edinburgh

## Objectives

Demographers are interested in the degree to which marriage is driven by prenuptial pregnancy within particular communities. To help answer this question, we present a novel method which links marriage certificates to birth certificates, where the birth-mother is the marriage-bride, considering only births which occur in the first seven months after a marriage.

## Approach

To identify prenuptial births we employed an unsupervised graph-based record linkage method to link birth and marriage certificates. We first extracted related groups of individuals: babies and their parents from birth certificates, and brides, grooms, and their parents from marriage certificates. To link births with marriages, we employed techniques to address challenges such as changing attribute values over time (such as names and addresses), the ambiguity of attribute values giving priority to rare names over common names, and different relationships encountered at different points in time (by applying temporal constraints). Based on the obtained links we then extracted prenuptial births.

## Results

Using two Scottish data sets containing a total of 38,451 births and 8,667 marriage certificates from the period 1861 to 1901 and employing different linkage thresholds we identified between 853 and 945 first birth-marriage links in the smaller (rural) data set, and between 2,165 and 2,232 links in the larger (urban) data set. In the rural data set, between 16.9% and 17.7% of these links were with birth less than 8 months after marriage (i.e. prenuptial births), where the corresponding ground truth contained 17.6% prenuptial births. For the urban data set, we identified between 51.3% and 51.4% prenuptial births. Our results show clear differences between rural and urban prenuptial pregnancies in 19th Century Scotland.

## Conclusion

We have presented an unsupervised graph-based record linkage method that compares attribute values of individuals and their relationships to link records based on the ambiguity of attribute values, attribute value changes, and temporal constraints. This linking process helps us to identify prenuptial births to help us understand the degree to which marriages may have been driven by pregnancy.

